# Relationship of serum ghrelin, leptin, resistin, adiponectin levels with nutritional status and inflammatory determinants in juvenile idiopathic arthritis

**DOI:** 10.1186/1546-0096-9-S1-P161

**Published:** 2011-09-14

**Authors:** Sanem Eren, Oya Sayın, Balahan Makay, Tuncay Küme, Erbil Ünsal, Filiz Kuralay, Nur Arslan

## Background

Juvenile idiopathic arthritis (JIA) is a chronic disease of childhood in which several proinflammatory cytokines are excessively secreted leading to anorexia and weight loss.

## Aim

To investigate the levels of ghrelin, a cytokine which is related to apetite and inflammation; adipose tissue cytokines (leptin, adiponectin and resistin) and proinflamatory cytokines (IL-6 and TNF-α) in JIA patients.

## Methods

40 JIA patients (19 active, 21 inactive patients) and 32 healthy controls were enrolled. Body weight, length, body mass index, and weight for heights were recorded.

After a 12-hour hunger period, blood samples were taken for ghrelin, IL-6, TNF-α, leptin, adiponectin, and resistin. The adipose tissue cytokines were proportioned to BMI in order to measure relative values.

## Results

There was not a significant difference between patients and the controls regarding the levels of ghrelin, TNF-α, IL-6 and resistin. There was a significant difference between patients and controls regarding the levels of relative adiponectin (p=0.041) and relative leptin (p=0.011). Regarding relative adiponectin, the difference between active group (0.21 ± 0.02) and inactive group (0.30 ± 0.08) was not statistically significant (p=0.507); however, there was a significant difference between controls (0.39 ± 0.04) and either active or inactive disease group (p=0.004 ve p=0.025, respectively). Relative leptin levels were statistically significant between control group and inactive disease group (p=0.000). We also compared the patients whose weight for height were 90-110 with healthy controls in order to make a comparement independent from the effects of adipose tissue on levels of leptin and adiponectin: levels of adiponectin were significantly lower in patients than controls (p=0.005), levels of leptin were significantly lower in patients than controls (p=0.000), levels of relative adiponectin were significantly lower in patients than controls (p=0.007) and levels of relative leptin were significantly lower in patients than controls (p=0.000).

## Conclusion

In this study, we showed that levels of relative leptin and adiponectin of JIA patients were lower than controls. This may be related to the inhibition of leptin and adiponectin production per unit of fat mass in the existence of disease. Further prospective studies including larger number of patients are required to demonstrate the effect of the disease and the treatments on adipokines in JIA patients.

